# Editorial: Cannabis and mental health: is it possible to predict safe use in the era of legalization?

**DOI:** 10.3389/fpsyt.2024.1468325

**Published:** 2024-09-10

**Authors:** Georgios A. Alevizopoulos, Artemis Igoumenou, Ioanna Vardakou, Aristomenis Alevizopoulos

**Affiliations:** ^1^ Department of Psychiatry, Agioi Anargyroi Hospital, National and Kapodistrian University of Athens, Athens, Greece; ^2^ Department of Psychiatry, University College London, London, United Kingdom; ^3^ Department of Toxicology, British American Tobacco, London, United Kingdom

**Keywords:** cannabis, mental health, legalization, safety, psychosis, cannabinoids and metabolites

## 1 Introduction

The legal status of cannabis is globally in flux. Exploratory and experimental work of some early states that have legalized both medical and recreational use provides an unparalleled opportunity for the more careful empirical work required to produce analysis of the full effects of currently controlled substances. The work largely presented here is inspired by the 2017 report from the National Academies of Sciences, Engineering, and Medicine on the health effects of cannabis and cannabinoids (1).

In this Research Topic we focus on the intersection of cannabis and mental health for several reasons. First, cannabis use has some well-established detrimental mental health effects. Heavy use of cannabis seems to be causally related to the onset of schizophrenia in some otherwise predisposed individuals, it has a small but significant effect on amotivation syndrome, it can exacerbate symptoms of bipolar disorder or bring on an episode of it, and it can intensify symptoms of PTSD (2). Furthermore, we focus on heavy users because they possess an increased likelihood of mental health issues significantly different than the general population of light users, and they have a higher prevalence of mental health issues than the general population.

The relationship between cannabis use and mental health has been studied for decades. These relationships are complex, and early research suggested that cannabis had many potential positive outcomes. Overall, the medical literature supports that medicinal and non-medicinal cannabis consumers alike may use cannabis to address underlying mental health disorders, with high rates of use suggested across a multitude of chronic health conditions (3).

The ability to predict the extent to which individuals use cannabis safely, especially among those who are at greater risk of experiencing its adverse effects, is central both to guiding educational efforts and to gauging the challenges that can be expected with wider legalization. Refining our understanding of these distinguishing features is of pivotal importance in the current era of legalization and recognized medical utility. Knowing the risks can help identify those for whom caution with everyday use is advisable, both for the medical uses that clearly are endorsed and for the opportunity to minimize drug-related harm among recreational users (4).

The societal trend toward legalization changes risk by altering the context and reducing stigma associated with usage. As such, there is already rising demand for methods that might differentiate safe users from those at risk of developing mental health problems consequent to use. These might range from providing individualized support to low-risk users, through a more stringent regulatory framework, to the possibility that predictive tools could be used to direct high-risk individuals away from cannabis use.

## 2 Focus on the contributions to this Research Topic

In this Research Topic of the Frontiers in Psychiatry we collected five research papers that address the relative risks towards a safer use of cannabis products when they become legally available for recreational reasons.

In their manuscript Baltes-Flueckiger et al. evaluate the impact of regulated cannabis access in pharmacies compared to the illicit market on problematic cannabis use, mental health, and physical health. Their study aims to inform addiction medicine and cannabis policies in Switzerland based on scientific evidence. The study “Weed Care” is focusing on its purpose, methodology, primary and secondary outcomes, and the potential implications of the research for addiction medicine and cannabis policy in Switzerland.

Sokratous et al. studied the attitudes of healthcare students towards cannabis use. The study findings offer insights for curriculum development, educational changes, and policy decisions regarding medical cannabis use in Cyprus. Majority of healthcare students supported medicinal cannabis use but reported a lack of knowledge. Their study exploring healthcare students’ attitudes, beliefs, and knowledge about medical cannabis use in Cyprus, shedding light on the need for education and curriculum adjustments in the healthcare field regarding medical cannabis.

With the rise of cannabis legalization, accidental and intentional exposure to cannabis-infused edibles has increased, especially among younger age groups, leading to concerns about hospitalizations. Conerney et al. in their study aim to identify and discuss risk mitigation strategies related to accidental consumption of cannabis edibles, providing guidance for policymakers. Regulatory restrictions on cannabis vary across legalized regions, making it challenging to identify best practices. The study examines practical aspects like compliance measures (e.g., regulatory audits) in managing risks associated with edibles. The authors are highlighting that edibles have a longstanding history predating recent regulatory efforts, emphasizing the need for effective risk management strategies to guide policymakers in regulating cannabis-infused products.

It is well known that synthetic cannabinoids share a significant proportion in cannabis market. In their contribution with a thorough review Heal et al. aim to evaluate abuse, dependence, and safety risks associated with individual cannabinoids in cannabis, including comparison with synthetic cannabinoids. They conclude that while cannabis presents some risks of abuse and dependence, they are comparatively lower than other substances. Non-psychoactive cannabinoids are not associated with intoxicating properties, but uncertainties regarding CBD’s influence on psychoactive effects warrant additional research. Synthetic cannabinoids, particularly potent CB1/CB2 agonists, pose a significant risk for abuse and harm.

Finally Binkowska et al. investigated products containing CBD that are gaining interest for their potential therapeutic benefits and positive impact on well-being and mental health. In the studied population they found that the users of CBD products for health and wellness purposes perceive potential health benefits. The authors encapsulate the CBD consumption patterns and the influence of various variables on its use, highlighting the perceived benefits of CBD for mental health and well-being, to enhance knowledge regarding CBD’s therapeutic efficacy and optimal dosing strategies.

## 3 Conclusions and future directions

As the prevalence of mental health disorders continues to rise globally, alongside a new era of marijuana use, it is more important than ever to understand how mental health symptoms and cannabis use can interact. Such an understanding is crucial for two reasons. First, there is reason to believe that the botanical-THC marijuana available today is significantly stronger than in the past, and its use can have a significant impact on mental health symptoms (5). This understanding is of particular significance to supporting youth that is the most vulnerable age-part of those who use cannabis (6). Despite the evidence of various recent reports, nearly 60% of youth do not think that frequent marijuana use carries any risks (7, 8).

These figures are particularly concerning as legal access to marijuana is seen by many youths as evidence that marijuana must be safe. The present Research Topic of studies suggests that research efforts could be redirected by looking at trends in cannabis use and factors important to youth as the laws governing marijuana change.
